# In psoriasis, levels of hope and quality of life are linked

**DOI:** 10.1007/s00403-014-1455-9

**Published:** 2014-02-25

**Authors:** Tomasz Hawro, Marcus Maurer, Marlena Hawro, Andrzej Kaszuba, Lidia Cierpiałkowska, Monika Królikowska, Anna Zalewska

**Affiliations:** 1 Department of Dermatology and Allergy, Charité – Universitätsmedizin Berlin, Chariteplatz 1, 10117 Berlin, Germany; 2 Psychodermatology Department, Medical University of Lodz, Pomorska 251, 92-213 Łódź, Poland; 3 Department of Dermatology, Paediatric Dermatology and Oncology, Medical University of Lodz, Kniaziewicza 1/5, 91-347 Łódź, Poland; 4 Institute of Psychology, Department of Health Psychology and Clinical Psychology, Adam Mickiewicz University in Poznan, Szamarzewskiego 89, 60-568 Poznań, Poland

**Keywords:** Psoriasis, Hope, Quality of Life, Psychotherapy, Positive psychology

## Abstract

Psychological resources such as hope have been suggested to positively influence quality of life (QoL) in chronic disorders. Here, we determined hope levels of psoriasis vulgaris in-patients and analyzed their relation to QoL. A total of 60 (29 male) patients were assessed for their QoL with a generic tool (WHOQOL-BREF) and a skin disease-specific instrument, the Dermatology Life Quality Index (DLQI). Hope levels were determined by use of the Basic Hope Inventory. We found a positive correlation between hope and all domains of WHOQOL-BREF (physical: *r* = 0.446, *p* = 0.000; psychological *r* = 0.464, *p* = 0.000; social *r* = 0.302, *p* = 0.019; environmental *r* = 0.480, *p* = 0000; and global *r* = 0.501, *p* = 0.000) and a negative correlation with DLQI (*r* = −0.281, *p* = 0.030) indicating higher QoL in patients with high hope. Hope was not correlated with disease severity or duration. Hope may play a substantial role in preventing QoL impairment in psoriasis. Psychotherapeutic interventions aimed at strengthening hope could improve QoL in this condition.

## Introduction

Psoriasis is a multifactorial skin disease, with a prevalence of 1.5–3 % in Western countries [7, 14]. The chronic and relapsing course of psoriasis, its resistance to therapy, the discomfort of therapy and the presence of skin lesions in exposed areas resulting in stigmatization all lead to significant quality of life (QoL) impairment. The impact of psoriasis on QoL was reported to be comparable to that of systemic life-threatening diseases such as arthritis, cancer, diabetes and cardiovascular disorders [20]. Recently, it was postulated that psychological factors and resources such as hope may play an important, moderating role on QoL impairment in patients with psoriasis [3, 12]. Hope is held to be an important protective factor in situations of threat and challenge [16]. According to Erikson, hope is an individual belief, that the world is orderly organized, rational and friendly to people, which appears in early childhood [4]. Hope has also been postulated to play a role in the adaptation to new situations and in the face of collapse of an existing order [28]. In other words, hope is thought to stimulate and maintain individuals’ constructive ways of coping with problems. In the face of personal loss, it helps to redefine aims and to search for new opportunities, thereby increasing chances to overcome crisis by finding an optimal solution.

It was shown that hope is positively correlated with QoL in the general population [2, 32], and hope has also been shown to have a positive role in chronic internal diseases, in terminally ill patients and their families [8, 11, 21]. Very recently, the relation of hope and QoL was first investigated in patients with psoriasis [27], and some domains of general QoL were suggested to benefit from high levels of hope. As of now, however, the link of hope and disease-specific QoL remains to be investigated and characterized in detail. We addressed this question and assessed and compared the levels of hope, of disease-specific as well as general QoL, and disease severity in hospitalized patients with psoriasis.

## Materials and methods

### Patient population and study features

Sixty patients with psoriasis vulgaris hospitalized at the Department of Dermatology, Pediatric Dermatology and Dermatological Oncology of the Medical University of Lodz participated in this cross-sectional study. The research was approved by the Ethic Committee of the Medical University of Lodz. The data were collected between January and June 2010. Consecutive patients admitted to the Department were invited to participate in the study on the day of their admission or on the following day. Inclusion criteria were age ≥18 years, ability to provide informed consent, ability to read and understand Polish, and the diagnosis of psoriasis vulgaris. Diagnosis of psoriatic arthritis was an exclusion criterion. Eight patients (two women) refused to participate in the study. Patients who signed the consent form were asked to fill the following questionnaires: Basic Hope Inventory (BHI)-12, Dermatology Life Quality Index (DLQI), WHOQOL-BREF quality of life questionnaire. The investigator dermatologist collected socio-demographic and clinical data, including assessment of Psoriasis Area and Severity Index (PASI).

### Psychometric/QoL instruments

Basic Hope Inventory-12, developed by Trzebinski and Zieba, was employed [28] to assess levels of basic hope, which is understood according to Erikson’s theory with further development by Trzebinski et al., as an individual’s belief in the world’s order, rationality and friendliness to people [4, 28]. BHI-12 is a self-assessment tool, consisting of 12 items. Each item is answered on a 5-point Likert scale. Three items act as buffers and are excluded from the analysis. The final outcome is a sum score for nine questions, which may lie between 9 and 45. The higher the score value, the higher the level of basic hope. Cronbach’s alpha test reliability was 0.70 [28].

Dermatology Life Quality Index is a self-administered 10-item questionnaire, developed by Finlay and co-workers as the first tool to measure skin disease-related QoL [5]. The validated, official Polish language version was used [26]. We used the total score of DLQI for our analyses. The total score can range from 0 to 30, with higher scores indicating lower QoL.

The WHOQOL-BREF is a generic QoL self-administered 26-item tool [23]. The instrument is based on the World Health Organization (WHO) definition, according to which QoL is: “an individual’s perception of their position in life in context of the culture and value systems in which they live and in relation to their goals, expectations, standards and concerns” [10, 30, 31]. Reliability Cronbach’s alpha coefficient of the Polish validation of the scale was 0.90. Responses to the items are scored on a 5-point Likert scale. Scores for particular domains range from 4–20. Higher scores denote better QoL. The scale consists of a physical domain (seven items), a psychological domain (six items), a social domain (three items) and an environment domain (eight items). The questionnaire also comprises two questions on individuals’ health status and QoL perception, analyzed together as “global QoL”, with a score range of 2–10.

### Clinical disease severity assessment

PASI allows for the evaluation of the clinical severity of the skin lesions in psoriasis [6]. The intensity of local erythema, induration and desquamation in a relationship to the percentage of the involved skin surface is recorded. The results may range from 0 to 72 with an increment of 0.1. Higher scores correspond to more severe disease.

### Statistical methods

Statistical analyses were performed using IBM SPSS 20 software. Normality of the distribution of the variables was tested with the Kolmogorov–Smirnov test. To compare two groups of variables, which were normally distributed, Student’s *t* test for independent samples was used (more than two groups: one-way ANOVA). Pearson′s correlation coefficient was used to assess correlation of basic hope with clinical and demographic variables and particular domains of QoL as well as overall QoL.

## Results

### Patients’ characteristics

The mean age of our patients was 46.8 years ± standard deviation (SD) of 14.2 years, range 19–74 years. The mean PASI score, i.e., clinical disease severity, was 15.2 ± 9.6 (SD) and the mean disease duration was 21.9 years ± 14.4 years (SD). These and further demographic and clinical characteristics as well as the levels for hope, general QoL and disease-specific quality of life are shown in Tables 1 and 2. The mean value of the basic hope in the analyzed group was 28.95 ± 4.49 (SD), range 14–39.

**Table 1:** Hope values, demographic and clinical variables in the studied group

Variables	Basic hope inventory score, mean ± standard deviation	*n* (%)
Gender		
Male	29.5 ± 3.9	29 (48.3)
Female	28.4 ± 5.0	31 (51.7)
Marital status		
Married	28.7 ± 4.9	26 (43.3)
Divorced	27.1 ± 4.2	10 (16.7)
Widow/widower	27.5 ± 2.3	6 (10.0)
Single	30.9 ± 4.2	18 (30.0)
Education		
Elementary school	29.7 ± 3.8	9 (15)
Secondary school	27.9 ± 3.9	19 (31.7)
High school	28.4 ± 5.0	23 (38.3)
University	32.0 ± 4.0	9 (15)
Employment status		
Employed	29.9 ± 4.4	34 (56.7)
Unemployed	28.0 ± 6.2	9 (15.0)
Disability pensioner	28.4 ± 2.7	8 (13.3)
Retired	25.9 ± 3.0	8 (13.3)
Student	36	1 (1.7)
Place of residence		
Town or city	28.6 ± 4.5	46 (76.7)
Rural area	30.3 ± 4.5	14 (23.3)
Family history of psoriasis		
Positive	28.6 ± 4.6	18 (30)
Negative	29.8 ± 4.3	42 (70)

**Table 2:** WHOQOL-BREF and dermatology life quality index (DLQI) values in the studied group

Quality of life	Mean	Standard deviation
DLQI	15.120	6.700
WHOQOL-BREF		
Physical domain	13.783	2.666
Psychological domain	12.212	2.532
Social relationship	13.152	3.699
Environment	12.728	2.212
Global quality of life	5.717	1.508

### High hope levels are correlated with better QoL

Hope levels were found to show a negative correlation with DLQI scores and a positive correlation with all WHOQOL-BREF domain scores, including global QoL, indicating that high hope levels are linked to better QoL in all analyzed areas (Table 3). In complementation of these primary correlation analyses, we stratified patients by their hope levels on the basis of the median value of hope (BHI score of 29). The scores of all WHOQOL-BREF domains as well as the global QoL score were found to be significantly lower in the group of patients with low levels of hope (Fig. 2). The difference in DLQI scores between patients with high and low hope levels was statistically not significant.

**Table 3:** Correlation between hope (BHI-12) and all investigated quality of life dimensions

	DLQI	WHOQOL-BREF				
		Physical domain	Psychological domain	Social domain	Environment domain	Global score
Hope (BHI-12)	*r* = −0.281; *p* = 0.030	*r* = 0.446; *p* = 0.000	*r* = 0.464; *p* = 0.000	*r* = 0.302; *p* = 0.019	*r* = 0.480; *p* = 0.000	*r* = 0.501; *p* = 0.000

*r* Pearson’s r coefficient, *p* significance level, *DLQI* *d*ermatology life quality index, *BHI* basic hope inventory

### Hope is linked to age but not to other patient features including disease duration or severity

**Fig. 1:**
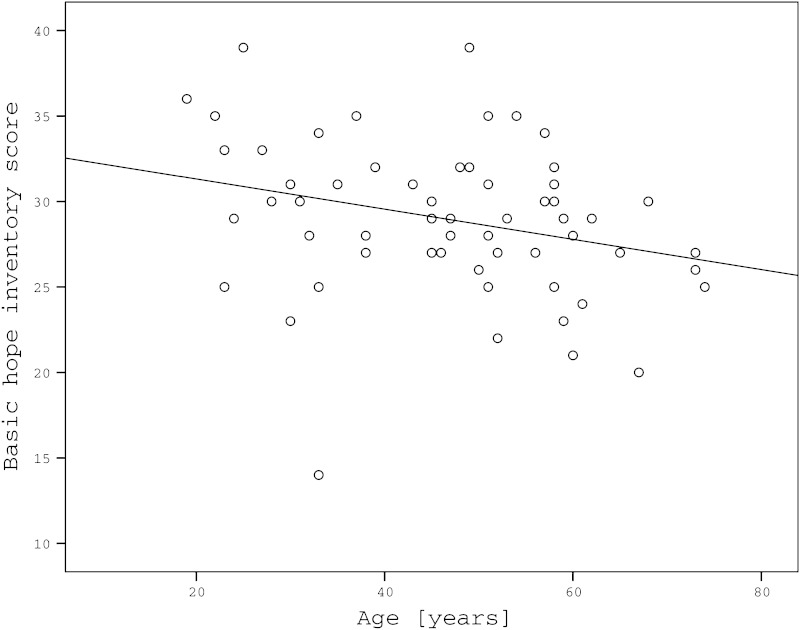
Correlation between age and basic hope inventory (BHI) score (Pearson’s *r* = −0.280; *p* = 0.030)

We found a weak negative correlation between the age of the patients and levels of hope (Pearson’s *r* = −0.280; *p* = 0.030, Fig. 1). There was no correlation of hope with disease severity (PASI) and duration. Also, there were no statistically significant differences in hope levels between patients grouped according to their gender, family history of psoriasis, marital status, level of education, place of residence or professional activity.

**Fig. 2:**
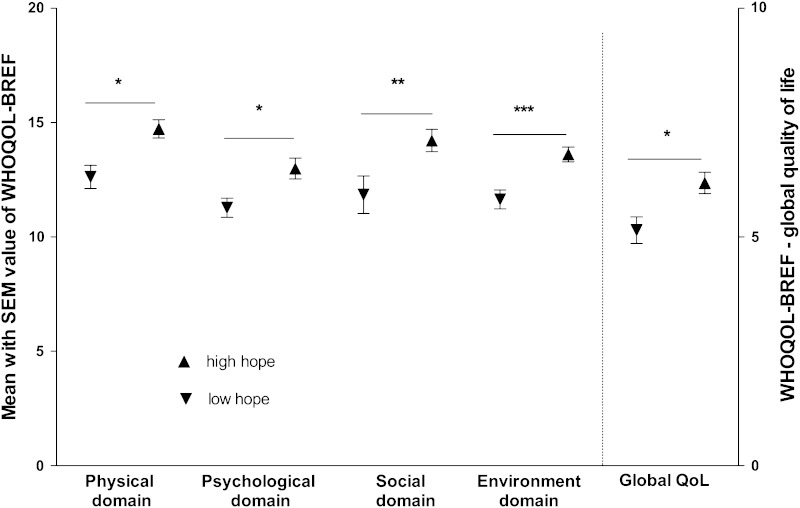
Psoriasis patients with low hope levels are more impaired in all domains of quality of life, SEM–standard error of the mean, * *p* < 0.01, ** *p* < 0.05, *** *p* < 0.001

## Discussion

Here, we show—to our knowledge for the first time—that hope in psoriasis patients is linked to health-related, skin disease-specific QoL. In addition, our results confirm recent evidence showing that hope is correlated to general QoL in patients with this disease [27]. Surprisingly, we also found that younger age is associated with higher hope levels in psoriasis, whereas disease severity or duration was not.

Our demonstration of an association of hope and health-related and skin disease-specific QoL raises two interesting questions: (1) Is the association of quality of life and hope specific for psoriasis? We believe it is not: recent studies have demonstrated that hope also correlates with a higher satisfaction with life in patients with serious mental illness as well as with lower levels of depression in cancer patients [19, 29]. (2) Are high hope levels in psoriasis the reason or the consequence of better health-related QoL? We believe that it is patients’ QoL that benefits from high hope, for three main reasons: (a) The hope instrument we used (BHI-12) is designed to assess basic hope, a belief about the world formed in early childhood. Hope according to the data of longitudinal studies remains stable at least over the follow-up period of up to 3 years [15, 17, 24, 25, 28]. (b) Hope as assessed employing a longitudinal approach and path analysis was shown to improve mood and anxiety, but mood and anxiety had no influence on hope [1]. (c) Basic hope levels have been shown to be similar in patients (including psoriasis patients) as compared to healthy controls [27]. Taken together, this argues that higher QoL in psoriasis patients is, at least in part, a consequence of high levels of basic hope. This view is supported by the notion that hope is regarded as a “stress buffer”, i.e., one of the psychological resources that help to cope with stress [8]. In line with this, psychological resources have previously been claimed to function as positive modulators of QoL in psoriasis [18]. Also, higher hope is known to correlate with more adaptive coping strategies, as shown for example, in blind US-American veterans [9]. Our results on hope and general QoL complement recently published observations [27]. These authors also used a general QoL tool (the Flanagan QoL Scale) and reported a correlation between basic hope in the physical and social domains of general QoL and a lack of any association with the remaining domains (personal development and fulfillment, recreation) and with overall QoL. In contrast, we found a positive correlation of hope levels with general QoL in all domains as well as with global QoL. Interestingly, the mean hope levels found by Szramka-Pawlak and co-workers (28.86) and by us (28.95) are very similar. Therefore, the differences in the results on correlation between hope and general QoL from the Szramka-Pawlak study and from our study are not due to differences in the hope levels, but rather due to differences of the two patient populations including the higher number of patients in our study as well as the use of different QoL instruments.

As of now, we cannot explain why higher levels of hope are associated with lower age in psoriasis. This phenomenon cannot simply be explained by the cumulative influence of the disease over time as hope did not correlate with psoriasis duration. Previously, increased QoL impairment and higher psychological distress were seen in a group of older psoriasis patients as compared to younger ones [22]. Available data on the stability of hope do not allow for conclusions on the stability of this construct over longer than 3 years. The correlation between hope and age detected in our study suggests that levels of hope may decrease over decades. Further studies on hope and other resources in larger numbers of patients with psoriasis are required to confirm and understand this finding.

### Limitations of the study

It must be noted that the cross-sectional character of the study does not allow for conclusions on the causal relationship between hope and quality of life. The considerations on causality were based solely on the literature data of longitudinal studies and on the theoretical basis of the hope concept. It is also possible that the observed correlation between hope and quality of life does not result from the fact that one of the two variables influences the other, but from involvement of a third variable influencing both, which could also be a methodological factor. It must be also noted that quality of life improvement may be attributed to many other factors, not only hope. Further limitations of the study are that only psoriasis in-patients were included and that the number of included patients was low. Thus, extrapolation of the results to the general population of patients with psoriasis must be performed very cautious.

Our results suggest that targeting hope in psoriasis patients may result in better QoL. On the one hand, hope according to Erikson’s concept is thought to be a relatively stable psychological component formed in the early developmental phase. On the other hand, hope is also seen as a cognitive structure, which can be expected to be susceptible to cognitive interventions. It was postulated that basic hope may change during life, especially in consequence of events that are critical to the individual, either destructive (decreasing hope) or constructive (increasing its level) [28]. Based on the latter concept, cognitive techniques aimed at increasing hope levels in cancer patients were developed [21] and an intervention based on hope theory was found to improve mood and increase hope levels in elderly patients with depression [13]. Thus, interventions aimed at strengthening of psoriasis patients’ hope, i.e. beliefs in the order and goodwill of the world, may help to improve their QoL.
